# EHMTI-0137. Headache as an initial clinical symptom of carotid artery dissection

**DOI:** 10.1186/1129-2377-15-S1-D59

**Published:** 2014-09-18

**Authors:** P Slankamenac, N Vukasinovic, Z Zivanovic, S Simic, I Divjak

**Affiliations:** 1 Department of neurology, Faculty of Medicine University of Novi Sad, Novi Sad, Serbia; 2 Department of neurology, Clinical Centre of Nis, Nis, Serbia

## Background

Carotid artery dissection (CAD) is main cause of stroke among young and middle-aged patients. Clinical presentation of CAD includes one side headache, pain on neck, often accompanied by Horner syndrome, and followed by cerebral ischaemia.

The aim of this study was to analyze the spectrum of clinical presentation in patients with CAD.

## Methods

This was a case series of 31 patients with CAD which were hospitalized from 2001 to 2014 at our department. The CAD was diagnosed in all cases by MRI, MRA and duplex sonography.

## Results

Average age of patients was 47,4 (28-59) years. From a total of 31 patients, there were 27 with unilateral and 4 with bilateral CAD. Facial and neck pain and Horner's syndrome were the only presenting symptoms in 6 patients; headache and visual disturbances in 2; headache and tinnitus in 1; facial pain, Horner's syndrome and contralateral sensorimotor deficit in 7; headache and contralateral sensorimotor deficit in 5; contralateral sensorimotor deficit in 10. CAD was spontaneous in 24 patients while in 7 was triggered by mild trauma. MRI revealed infarction in 22 patients. Between patients without brain infarction, 6 patients presenting with facial and neck pain and Horner's syndrome, 2 with headache and visual disturbances and 1 with headache and tinnitus. Good outcome (defined as modified Rankin score of 0-2) was seen in 28 patients (90,3%).

## Conclusion

CAD was associated with headache in 21 patients (67,7 %). However, the clinical presentation of CAD is variable and can be similar to other etiology of stroke.

No conflict of interest.

